# Nanoencapsulation of Bacteriophages in Liposomes Prepared Using Microfluidic Hydrodynamic Flow Focusing

**DOI:** 10.3389/fmicb.2018.02172

**Published:** 2018-09-12

**Authors:** Salvatore Cinquerrui, Francesco Mancuso, Goran T. Vladisavljević, Saskia E. Bakker, Danish J. Malik

**Affiliations:** ^1^Chemical Engineering Department, Loughborough University, Loughborough, United Kingdom; ^2^Advanced Bioimaging Research Technology Platform, University of Warwick, Coventry, United Kingdom

**Keywords:** bacteriophage therapy, *E. coli*, intracellular infections, liposome fabrication, microfluidics, nanoencapsulation, *S. aureus*

## Abstract

Increasing antibiotic resistance in pathogenic microorganisms has led to renewed interest in bacteriophage therapy in both humans and animals. A “Trojan Horse” approach utilizing liposome encapsulated phages may facilitate access to phagocytic cells infected with intracellular pathogens residing therein, e.g., to treat infections caused by *Mycobacterium tuberculosis, Listeria, Salmonella*, and *Staphylococcus* sp. Additionally, liposome encapsulated phages may adhere to and diffuse within mucosa harboring resistant bacteria which are challenges in treating respiratory and gastrointestinal infections. Orally delivered phages tend to have short residence times in the gastrointestinal tract due to clinical symptoms such as diarrhea; this may be addressed through mucoadhesion of liposomes. In the present study we have evaluated the use of a microfluidic based technique for the encapsulation of bacteriophages in liposomes having mean sizes between 100 and 300 nm. Encapsulation of two model phages was undertaken, an *Escherichia coli* T3 podovirus (size ~65 nm) and a myovirus *Staphylococcus aureus* phage K (capsid head ~80 nm and phage tail length ~200 nm). The yield of encapsulated T3 phages was 10^9^ PFU/ml and for phage K was much lower at 10^5^ PFU/ml. The encapsulation yield for *E. coli* T3 phages was affected by aggregation of T3 phages. *S. aureus* phage K was found to interact with the liposome lipid bilayer resulting in large numbers of phages bound to the outside of the formed liposomes instead of being trapped inside them. We were able to inactivate the liposome bound *S. aureus* K phages whilst retaining the activity of the encapsulated phages in order to estimate the yield of microfluidic encapsulation of large tailed phages. Previous published studies on phage encapsulation in liposomes may have overestimated the yield of encapsulated tailed phages. This overestimation may affect the efficacy of phage dose delivered at the site of infection. Externally bound phages would be inactivated in the stomach acid resulting in low doses of phages delivered at the site of infection further downstream in the gastrointestinal tract.

## Introduction

The widespread inappropriate use of antibiotics in both humans (clinical medicine) and animals (livestock industry) worldwide has led to an acceleration in the emergence and global spread of multidrug antibiotic resistant bacterial clones (Morgan et al., 2011). The problem of antibiotic resistance is a complex one requiring global coordination for antibiotic stewardship to preserve the efficacy of current treatments. In much of the world outside Europe and North America, lifesaving antibiotics are sold without a prescription or oversight by health professionals (Laxminarayan et al., 2013). In the period between 1940 and 1962, 20 new classes of antibiotics were introduced to the market; however, since 1962, there has been a discovery void with only two new classes reaching this stage (Coates et al., 2011). The speed of development of resistance has been faster than the rate of discovery (Kelly et al., 2016). The substantial public health threat from antibiotic resistance includes jeopardizing effectiveness of treatments in modern medicine from minor elective surgeries to cancer therapy.

The ESKAPE pathogens (*Enterococcus faecium, Staphylococcus aureus, Klebsiella pneumoniae, Acinetobacter baumannii, Pseudomonas aeruginosa*, and *Enterobacter* species) represent a key group of commonly occurring Multi-Drug Resistant organisms at the heart of the antibiotic resistance crisis (Schooley et al., 2017). Initiatives to develop new therapeutic approaches with novel mechanisms of activity against MDR bacterial pathogens include the potential use of lytic bacteriophages (Summers, 2001; Czaplewski et al., 2016). Lytic bacteriophages (phages) are viruses that infect and kill bacteria, and they represent a promising approach to targeting bacterial infections in a treatment known as phage therapy (Alisky et al., 1998; Abedon, 2009; Abedon et al., 2011; Allen et al., 2014). The specificity of bacteriophages and their potential role in maintaining healthy microbiota makes them an attractive alternative to employing antibiotics. Technical advances are reducing the cost, processing and sequencing times of next-generation sequencing (NGS), thereby allowing rapid culture independent identification of disease causing bacteria (Toma et al., 2014). These developments increase the opportunities for using narrow spectrum antibiotics where the infection causing bacterial agent is known, thereby opening-up the possibility of employing phages for therapeutic purposes (Malik et al., 2017). A number of recent studies in animals and humans have been carried out to investigate the clinical safety and therapeutic or prophylactic efficacy of phages against *P. aeruginosa, S. aureus, A. baumannii, Escherichia coli*, and *Salmonella enteritidis* (Wright et al., 2009; Lim et al., 2012; Sarker et al., 2016; Schooley et al., 2017).

Limitations regarding the broad utility of phage therapy for the treatment of bacterial infections include narrow host ranges of individual phages and bacterial host resistance leading to phage resistant mutant. There is some debate as to whether bacteriophages would be able to diffuse across eukaryotic cell membranes killing intracellular bacteria infecting macrophages and other eukaryotic cells. Some recent *in vitro* studies have shown free phage entry into macrophages and other non-phagocytic eukaryotic cells however, the mechanisms of entry remain unclear (Nieth et al., 2015b; Zhang et al., 2017). Intracellular phage entry into eukaryotic cells has been explained to occur either through phagocytosis of phage infected bacteria or via bacterial induced endocytosis (Finlay, 1997). Important multi-drug resistant intracellular pathogens include those causing chlamydia, salmonella, staphylococcal, and mycobacterial infections, as well as infections due to *Brucella abortus, Burkholderia cenocepacia, K. pneumoniae, Legionella pneumophilia*, and *Listeria monocytogenes* (Nieth et al., 2015a; Singla et al., 2016b). Use of phage cocktails permits broadening of the host range whilst judicious selection of phages making-up the cocktail targeting different receptors tends to reduce the risk of resistance arising in host bacteria due to random mutations (Tanji et al., 2005; Denou et al., 2009; Yen et al., 2017). In chronic infections phage treatment may be compromised by the adaptive host immune response (anti-phage antibodies) leading to clearance of delivered phages lowering the phage titer at the site of infection resulting in poor efficacy of phage therapy (Dabrowska et al., 2005). Another considerable challenge is associated with accessibility of phages to a wide range of pathogenic bacteria residing within mucosa and biofilms including highly resistant strains of *Bordetella pertussis* (whooping cough), *E. coli* (diarrheas, meningitis, urinary tract infections), *Helicobacter pylori* (ulcers, gastritis), *Neisseria gonorrhoea* (gonorrhoea), *Neisseria meningitidis* (meningitis), *P. aeruginosa* (infections in people suffering from cystic fibrosis), *S. aureus* (wound associated infections e.g., diabetic foot ulcers), streptococci (meningitis, otitis media, pharyngitis, scarlet fever), etc.

Encapsulation of phages in liposomes may address some of the challenges associated with the development of effective phage therapy discussed above. Liposomes are composed of phospholipids, which self-assemble and self-enclose to form spheres of lipid bilayers with an inner aqueous core which may be designed to contain therapeutic agents such as phages. Liposomes may aid in shielding phages from the action of the reticuloendothelial system thereby increasing the length of time of circulation of phages instead of having to find long-circulating mutant phage strains (Merril et al., 1996). A previous study demonstrated that non-virulent *Mycobacterium smegmatis* and *Mycobacterium avium* transiently infected with the lytic phage TM4 were able to deliver the phage into *Mycobacterium tuberculosis* infected macrophages in a “Trojan Horse” approach (Broxmeyer et al., 2002). In a similar manner, liposome encapsulated phages may permit phage access to intracellular pathogens for the treatment of serious infections, such as those caused by *M. tuberculosis, S. aureus*, and *E. coli* (Nieth et al., 2015b).

Liposomes have been used to encapsulate a vast variety of cargos, like hydrophilic and hydrophobic drugs, proteins, living cells, nanoparticles, quantum dots, plasmid DNAs (Pattni et al., 2015). Ideally, liposome encapsulation techniques should allow full control over liposome formulation composition (e.g., for regulatory safety compliance) as well as control over vesicle size and size distribution which are important factors as they affect the encapsulated dose as well as *in vivo* circulation times and pharmacokinetics of the encapsulated active ingredient and its biodistribution (Sawant and Torchilin, 2012b). The liposome production process needs to be scalable and have a low environmental impact (Torchilin, 2005). Since their discovery in the 60s, several methods for the preparation of liposomes have been developed (Sawant and Torchilin, 2012a). However, classical techniques (e.g., thin film hydration, sonication, solvent dispersion, detergent removal, dilution) as well as sophisticated/innovative ones (e.g., use of supercritical fluids) do not completely fulfill the requirements for an ideal encapsulation process and lack adequate control over liposome production. The choice of the manufacturing technique is constrained by specific requirements and is quite often a compromise. Recently, microfluidic approaches have been used to address some of the constraints of other liposome production methods (van Swaay and deMello, 2013). Microfluidic devices offer many advantages over traditional liposome fabrication methods, such as control over the average size of liposomes, narrow particle size distribution, portability, integration and automation possibilities, and small reagent consumption thereby allowing rapid screening of formulations and production parameters. Liposome size and size distribution affect crucial characteristics including cargo release profile, loading capacity, biodegradation rate, biodistribution, and liposome stability over time.

There are relatively few published studies on phage encapsulation in liposomes (Nieth et al., 2015a; Singla et al., 2015, 2016a,b; Chibber et al., 2017; Leung et al., 2018). Recent animal studies in chickens have shown that liposome encapsulated *Salmonella* phages were significantly more stable upon exposure to stomach acid and showed longer intestinal retention times in comparison with free phages and this resulted in better treatment outcomes (Colom et al., 2015). Liposome encapsulated *K. pneumoniae* phage delivered via intraperitoneal injection remained in the systemic circulation of mice longer than the corresponding free phages (Singla et al., 2015). Liposomal nanoparticles (NPs) may be able to penetrate deep into the intestinal mucosa to reach infection sites, thereby improving the efficacy of phage therapy by targeting pathogenic bacteria residing in these difficult to access niches (Takeuchi et al., 2005). The residence time of NPs has been found to be longer compared to larger particles. For instance, chitosan coated sub-micron liposomes showed longer residence times in the GI tract than larger multilamellar vesicles attributed to their high penetration ability into the mucosa (Takeuchi et al., 2005; Thirawong et al., 2008).

The aim of this study was to investigate the use of a novel co-flow microfluidic glass capillary device for the encapsulation of bacteriophages in sub-micron sized liposomes. Previous studies have typically employed a thin-film hydration method for liposome preparation which does not afford precise control over the resulting liposome size and phage encapsulation. The effects of lipid composition, phage titer and experimental procedures related to encapsulation of two model phages, an *E. coli* T3 podovirus and a myovirus *Staphylococcus* phage K, have been investigated.

## Materials and methods

### Bacteria and phage strain and propagation

Bacterial host strains used were *S. aureus* ATCC 19685 and *E. coli* ATCC 11303 strain B. Both strains were sourced from LGC standards, United Kingdom (UK). *S. aureus* bacteriophage K ATCC 1985-B1 (family *Myoviridae*) and *E. coli* bacteriophage ATCC® 11303-B3 (family *Podoviridae*, T3) were also sourced from LGC standards, UK.

*S. aureus* bacterial host strain and phage were propagated in liquid culture using Brain Heart Infusion (BHI, Oxoid, UK). Luria Broth (LB, Oxoid, UK) was used to grow *E. coli*. For *S. aureus* phage propagation, a single colony from a streaked overnight culture on a BHI agar plate was used to inoculate fresh BHI broth and left shaking overnight at 37°C. The culture was diluted to O.D. 0.05 and regrown to log phase between 0.2 and 0.3 O.D. (λ = 600 nm) for all phage work. To evaluate actual MOI a 10 μl sample was withdrawn and serially diluted to measure the CFU/ml of the sample at the point prior of addition of a known quantity of phage. Previously, growth curves were measured where both the O.D. and corresponding CFU/ml measurements were obtained under identical growth conditions; the measurements were reliably repeatable (data not shown). The correspondence between growth curve CFU/ml and O.D. values gave us confidence that using O.D. values between 0.2 and 0.3 for both *S. aureus* and *E. coli* corresponded to ~10^8^ CFU/ml prior to phage addition. At this point bacteriophages of known titer were added to the culture at a multiplicity of infection (MOI) of ~0.01. Once the culture had cleared (~6 h later) the lysate was centrifuged at 2,000 *g* for 15 min at 4°C and the supernatant filtered using a 0.22 μm pore size in-line syringe filter (Millipore, USA). Hundred kilo Daltons of MWCO tangential flow ultrafiltration cassettes (Merck Millipore, UK) were used for exchange of lysate media with SM buffer (50 mM of Tris-HCl, pH of 7.5, 100 mM NaCl, 10mM MgSO_4_, all chemicals purchased from Fisher Scientific, UK) and if needed to concentrate the phage titer. All phage samples were stored in SM buffer at 4°C until further use.

To titer the phage samples, phage solutions were serially diluted and spotted (six replicates) on a double layer agar plate (Adams, 1959). The plate was dried near a flame and then incubated overnight at 37°C. On the following day plaques were counted to determine the phage titer and expressed as plaque forming units PFU ml ^−1^.

### Reagent chemicals

Phospholipid DSPC (1,2-distearoyl-sn-glycero-3-phosphocholine) was purchased in dry powder form from BACHEM (Bubendorf, Switzerland) and was used without any further purification. Cholesterol ≥99%, Chloroform anhydrous ≥99%, Isopropanol anhydrous 99.5%, and Triton X-100 were purchased from Sigma-Aldrich, UK. Ethanol 99.5% (extra dry) was purchased from Fisher Scientific, UK.

### Co-flow microfluidic device fabrication

**Figure 1:**
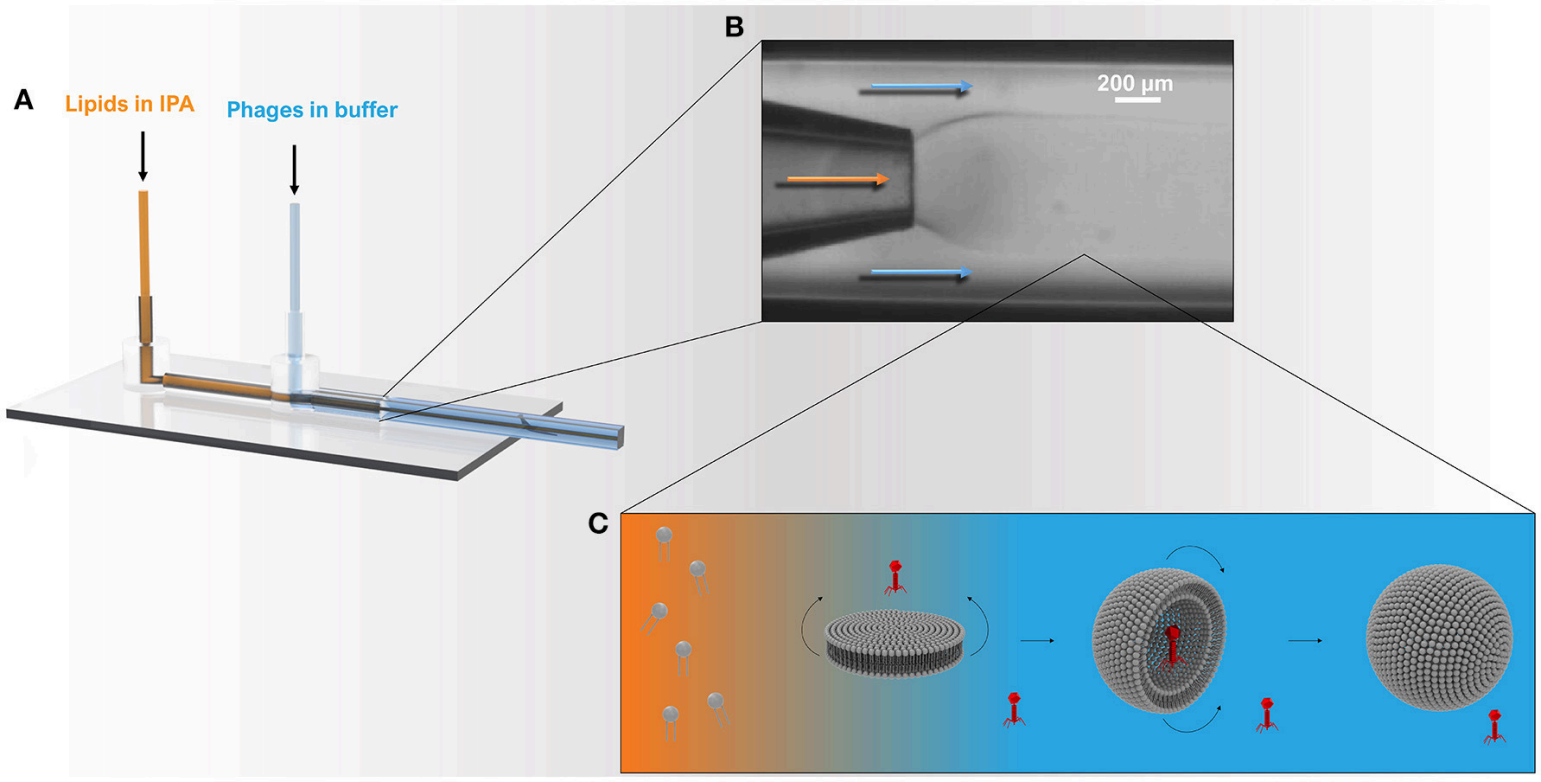
Schematic showing the microcapillary micromixing device for the production of liposomes. **(A)** 3D model of the co-axial glass capillary device. Solutions were pumped using two microfluidic pumps using a total flow rate of ~1 ml h^−1^ and a flow rate ratio (FRR) of aqueous to organic phase (Q_a_:Q_o_) of 2:1. **(B)** The round capillary was inserted into a square capillary that delivered the aqueous phase containing phages. The internal round capillary delivered the lipid dissolved in IPA solution. **(C)** Once the organic phase mixes with the aqueous phase, the phospholipids experience a highly polar environment that leads to self-assembly of lipids into bilayer discs. The latter grow in the radial direction by collision with other discs or through addition of phospholipid molecules from solution. When the disc bending energy is overcome by the energy gain when the edge around the bilayer disc is removed, the planar bilayer closes upon itself resulting in a spherical structure entrapping the surrounding solution and any phages in the vicinity of the bilayer.

The co-flow microfluidic (MF) device used for liposome formation was fabricated in-house following the procedure reported earlier (Vladisavljevic et al., 2014). The main body of the MF device consisted of two coaxial borosilicate glass capillaries glued onto a microscope glass slide. To assemble the device, a round tapered-end borosilicate glass capillary (Intracel, Royston, UK) with a 1 mm outer diameter, 0.58 mm inner diameter, and 100–200 μm orifice diameter was inserted halfway into an outer square glass capillary (1 mm inner dimension, AIT Glass, Rockaway, US) and aligned (see Figure 1). A P-97 Flaming/Brown micropipette puller (Sutter Instrument Co., USA) was used to produce a sharp capillary tip of about 20 μm in diameter. The orifice size of inner capillary was then increased by sanding the tip against sand paper until the orifice with a required size and smooth rim was obtained. The orifice size was controlled using a Narishige's MF-830 microforge microscope (Intracel Ltd., Stephenson, UK). Two syringe needles (BD Precisionglide®, Sigma–Aldrich, O.D. 0.9 mm) and PTFE tubing (I.D. 0.8 mm) were used to deliver the organic phase and the aqueous phage solution.

### Generation of liposomes

Liposomes were produced using the MF device described above (Figure 1) and the method of direct co-flow alcohol injection (Vladisavljevic et al., 2014). Lipid solutions (with different DSPC:cholesterol ratios) were prepared by dissolution of lipids in chloroform in a round bottom flask. The lipids were dried using a rotary evaporator for 2 h to obtain a thin film, and subsequently further dried overnight under vacuum. Isopropanol (IPA) was used to solubilize the film of lipids to a final concentration of 10 mg ml^−1^. The organic lipid solution and the aqueous buffer solution were delivered, respectively, through the inner tapered round capillary and the outer square capillary by means of Vici Model M6 microfluidic pumps (Valco Instruments Co. Inc.,). The flow rates of the organic phase, Q_o_ and the aqueous phase, Q_a_ were adjusted to control the degree of hydrodynamic focusing and the width of the central stream, thus controlling the IPA/buffer micromixing process. The flow rate ratio (FRR = Q_a_:Q_o_) between the outer aqueous phase and the inner organic phase of 2:1 was used. The MF device was immersed in a water bath and allowed to reach thermal steady state under experimental conditions to ensure the experiments were performed under constant temperature. The IPA solution was filtered through 0.1 μm PVDF syringe filters (Fisher Scientific, UK) prior to pumping through the MF device. Liposomes were collected in sterile glass vials (Fisher Scientific, UK).

### Nanoencapsulation of bacteriophage in vesicles

Bacteriophages were encapsulated in liposomes as per the procedure outlined above. Purified concentrated bacteriophages in SM buffer were prepared at titers >10^11^ PFU/ml. This concentrated phage stock was diluted to the appropriate working concentration by mixing with SM buffer prior to use in the MF device if lower concentrations of phage were used for encapsulation (working concentrations were varied as needed in the range 10^9^-10^10^ PFU/ml). The aqueous phage solution was delivered through the outer capillary, while the organic lipid solution was injected through the inner capillary.

### Determination of encapsulated bacteriophage K titers

#### Separation of free *S. aureus* phage K from liposome suspensions

The *S. aureus* phage K product stream collected from the MF device containing a mixture of free and encapsulated phages was dialyzed (Visking Dialysis Membranes, Molecular Weight Cut-off 3,500 Da) overnight against SM buffer (pH 7.5) to remove residual isopropanol. Free K phages were separated from liposomes using a 0.22 μm MidGee hoop ultrafiltration hollow fiber tangential flow cartridge with a 1 ml hold-up volume (GE Healthcare, UK). The membrane flux was 2 l/m^2^.h and run for 8 h until no further change in retentate phage titer was recorded. The liposomes were retained in the retentate and free phage removed in the permeate.

#### Removal of liposome bound *S. aureus* phage K

To evaluate the binding of free K phages to liposomes, empty liposomes (formulation described previously) at a concentration of 10^10^ liposomes/ml were gently mixed with phage K solution (final concentration in liposome solution ~10^10^ PFU/ml). The total volume was 1 ml. The empty liposomes were left in contact with K phages for 15 min at either 4, 25, or 37°C to allow the phages to adsorb to the liposomes. Liposomes were separated from unbound free phages by centrifugation at 13,000 g for 3 min and phage titer in the supernatant was measured using double overlay plaque assay technique.

To evaluate the inactivation of liposome bound phages, empty liposomes were left in contact with K phages for 15 min at 37°C to allow the phages to adsorb to the liposomes (as described above). The solution was then dialyzed against SM buffer (pH 2.75) for 60 min. The solution was subsequently neutralized with NaOH (0.1 M) followed by measurement of the phage titer by double overlay plaque assay technique. All phages were rendered unviable.

Inactivation of phage K adsorbed to the liposome bilayer membrane was undertaken to evaluate the encapsulated phage K titer. Liposomes containing K phages were exposed to pH 2.75 to inactivate the liposome adsorbed phages. Phage K were prepared in a pH 5.5 SM buffer and were encapsulated using the microfluidic device as per the procedure described above. The lower pH of the buffer was used to reduce the proton concentration gradient and therefore the proton flux across the membrane to minimize pH change within the liposome compartment (Deamer, 1987). Encapsulation at pH 7.5 was found to result in complete inactivation of phage K in the liposomes exposed to pH 2.75 hence lowering of the internal pH to pH 5.5 was undertaken which resulted in viable phage recovery. Phage K titer was stable over a period of 24 h in pH 5.5 buffered solution. Phage K was completely inactivated (10 log reduction) at pH 4 (data not shown).

Free phage K (titer ~10^10^ PFU/ml) was dialyzed against SM buffer (pH 2.75) for 60 min. The solution was subsequently neutralized with NaOH (0.1 M) followed by measurement of the phage titer by double overlay plaque assay technique. All free phages were rendered unviable.

The liposome samples containing encapsulated phages was dialyzed against SM buffer (pH 2.75) for 60 min, subsequently neutralized with NaOH (0.1 M) followed by measurement of the phage titer by double overlay plaque assay technique to ensure that all remaining free phages were rendered unviable. The pH in the dialysis bag was measured and attained at pH 2.75 within 30 min. Encapsulated phages were then released by disrupting the liposomes with Triton X-100 according to a previously described protocol (Patra et al., 1998). The final concentration of both Triton X-100 and (NH_4_)_2_SO_4_ in the liposomal dispersion was 5 mM. The sample was left in a water bath at 40°C for 1 h and the phage titer after disruption of the liposomes was assessed by plaque assay. We found that at 40°C there was no change in phage titer for both phage K and T3 phages (data not shown).

### Determination of encapsulated bacteriophage T3 titers

#### Separation of free *E. coli* T3 phages from liposome suspensions

*E. coli* T3 bacteriophages were encapsulated in liposomes as per the procedure outlined above. The titer of phages in the liposome collection vial was evaluated to give an indication of the inactivation of T3 phages due to exposure to IPA. Free T3 phages were separated from liposomes by centrifugation at 9,000 × *g* for 5 min. The liposome pellet was resuspended in SM buffer at pH 7.5, this procedure was repeated three times to remove free T3 phages and titer of phages measured. Encapsulated phages were then released by disrupting the liposomes with Triton X-100 according to a previously described protocol.

To evaluate the effect of phage T3 titer on encapsulation yield, three different initial titers of phage T3 (10^9^, 10^10^, 10^11^ PFU/ml) were used for encapsulation. The process of encapsulation was identical to the method described above. Following separation of free phages from liposomes by centrifugation (details above), encapsulated T3 phages were released by disrupting the liposomes with Triton X-100 (described above) and encapsulated phage titer was assessed by plaque assay.

### Characterization

#### Liposome size and size distribution

A NanoSight LM10 (Malvern Instruments Ltd., UK) using nanoparticle tracking analysis (NTA) was used to determine the average size and size distribution of both liposomes and bacteriophages. NTA measurements were performed in a sample chamber equipped with a 640 nm laser to track the NPs. Typically, a 10 μl aliquot was taken from each sample and diluted 10^2^-10^3^ fold in order to achieve a particle concentration of 10^7^-10^10^ particles ml^−1^. The sample was injected into the sample chamber using a sterile syringe and sample flow was maintained through the chamber until all air bubbles were removed. The temperature was registered with a thermometer (RTD Pt100, OMEGA, UK) and temperature correction was carried out. The software used for capturing and analyzing the data was NTA 3.0 (Malvern Instruments Ltd., UK). Data for each sample was captured over a period of 60 s and each measurement was repeated five times. The focus was set to achieve a uniform perfect spherical particle view. Before capturing the video, the camera had to set-up to ensure all the particles in the sample were clearly visible with no more than the 20% saturation. The single gain mode was used throughout the whole measurement process. Statistical analysis was carried out using the NTA software.

### Cryo-transmission electron microscopy (cryo-TEM)

An 8 μl aliquot of sample was pipetted onto a carbon coated copper grid (HC300Cu, Holey Carbon film on Copper 300 mesh, EM Resolutions, UK). Excess liquid was blotted away with filter paper (Whatman number 1) and the grid was plunge-frozen in a liquid mixture of ethane/propane cooled by liquid nitrogen. The sample was then kept at liquid nitrogen temperatures throughout the analysis. TEM images were taken on a JEOL 2200FS TEM at 200 keV using a Gatan K2 Summit and Gatan 914 cryo-holder.

## Results

**Figure 2:**
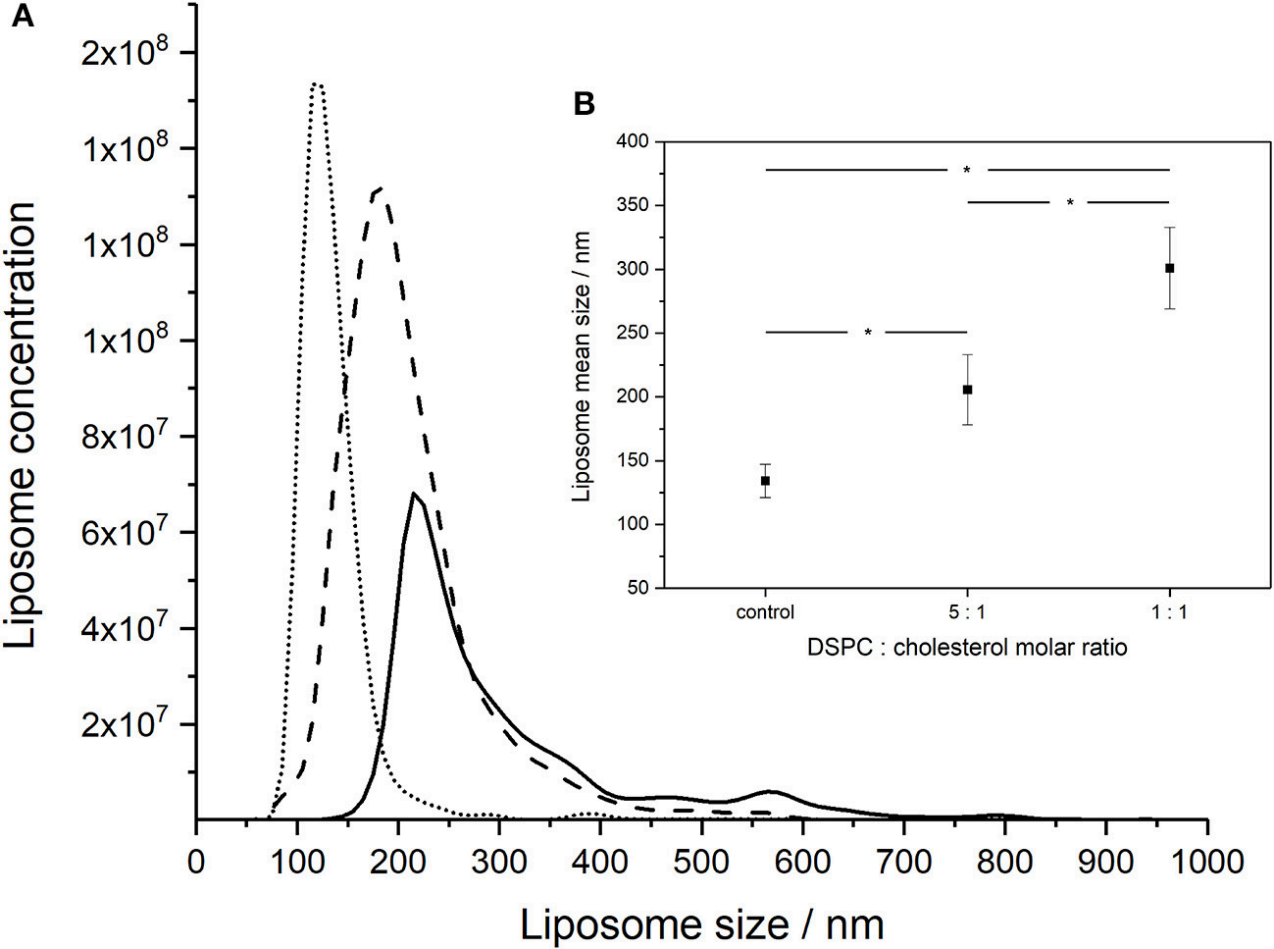
NanoSight measurements of the size distributions of liposomes formed using different DSPC:cholesterol molar ratios: **(A)** DSPC only (dotted line), 5:1 DSPC:cholesterol molar ratio (dashed line), and 1:1 DSPC:cholesterol molar ratio (solid line). Liposomes were prepared at room temperature (~20°C) using aqueous to organic phase volumetric flow rate ratio FRR of 2:1 and the total volumetric flow rate of ~1 ml h^−1^. **(B)** Inset showing average mean sizes, error bars indicate one standard deviation. *Indicates significance (*p* < 0.05) using 2-sample *t*-test.

Increasing the concentration of added cholesterol in the liposome formulation resulted in an increase in the liposome mean size and broadening of the size distribution (Figure 2A). NTA measurements indicated that the mean size of the liposomes increased with the amount of added cholesterol from 134 ± 13 nm for pure DSPC, to 206 ± 28 nm for a DSPC:cholesterol molar ratio of 5:1 and finally 301 ± 32 nm for a DSPC:cholesterol molar ratio of 1:1.

**Figure 3:**
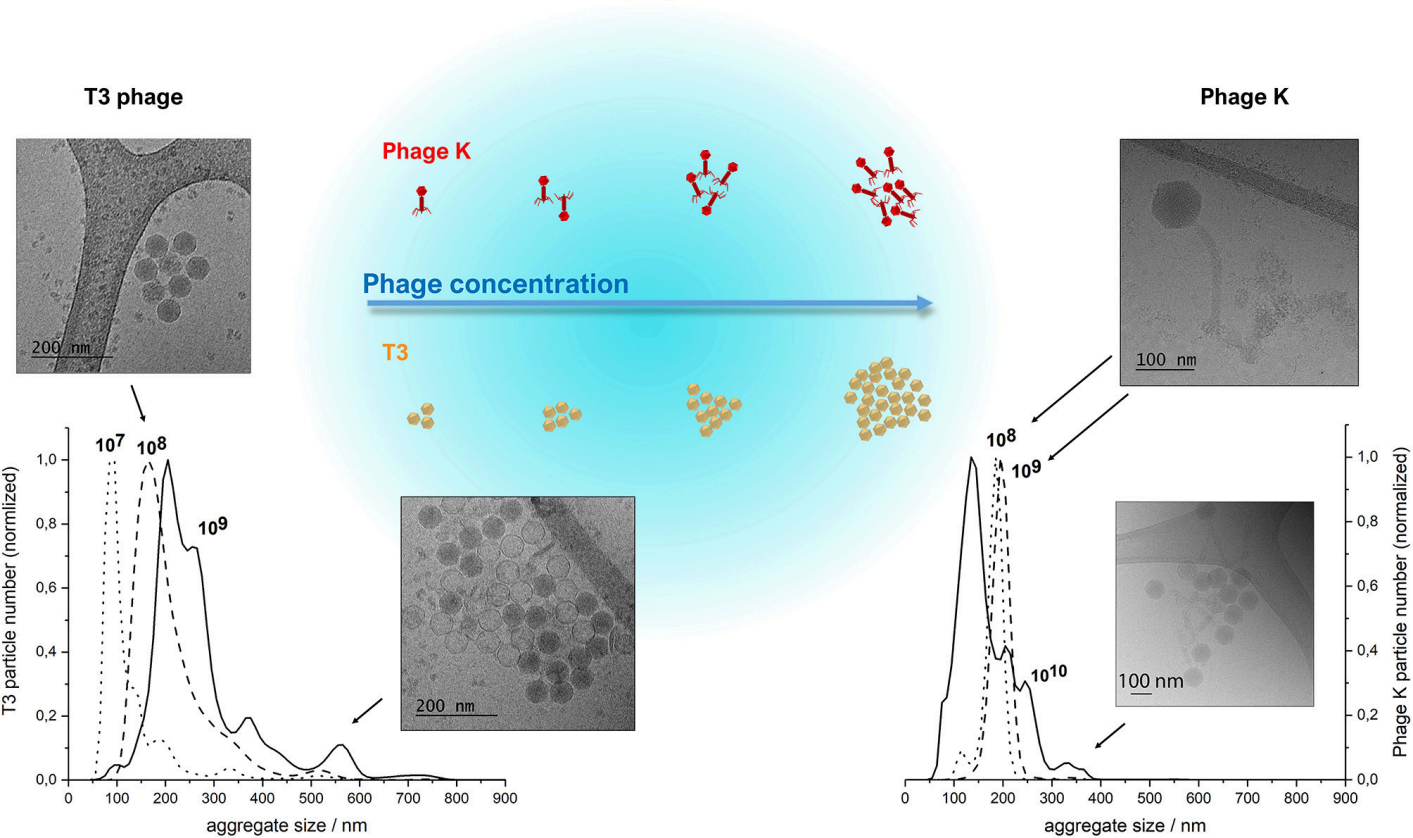
NanoSight NTA measurements of size distributions of purified T3 phage (left) and phage K (right), respectively, at 10^7^, 10^8^, 10^9^ and 10^8^, 10^9^, 10^10^ PFU/ml. Insets show CryoTEM images of observed phage aggregates and the schematized depiction of phage cluster sizes as a function of phage titer. Some heads on the lower CryoTEM images are transparent (empty), probably due to denaturation of DNA during encapsulation or loss of DNA during CryoTEM imaging.

The morphology and size of phage T3 (*Podoviridae* family; phage dimensions ~65 nm) and phage K (*Myoviridae* family, capsid head ~80 nm and phage tail length ~200 nm) were visualized using cryo-TEM (Figure 3). Concentrated purified phages in SM buffer were initially prepared at high phage titers of ~10^10^ PFU ml^−1^ on the premise that there would be an increasing likelihood of phage encapsulation in liposomes resulting in higher phage encapsulation yield, but high phage titers promoted phage aggregation. Phage aggregation into larger clusters was observed for T3 at relatively low phage concentrations, as low as 10^7^ PFU ml^−1^. However, phage K showed noticeable aggregation only when phage titers were ~10^10^ PFU ml^−1^ (Figure 3). T3 phages were more susceptible to aggregation due to the compact shape of their tail-free heads. Phage K began to aggregate only at high concentrations due to steric hindrance imposed by the long tails of these phages.

The clusters of both phages were found to be stable at high phage concentrations irrespective of ionic strength of the solution, since the presence of various salts at different concentrations did not affect the size of the clusters (data not shown). Dilution of phages resulted in a reduction in the average cluster size suggesting the aggregation process was reversible (Figure 3). The average size of phage clusters is dictated by the dynamic equilibrium between the rate of aggregation due to attractive Van der Waals forces acting between the phages and the rate of disaggregation due to their thermal motion. The rate of phage aggregation increases with an increasing rate of phage collisions in the solution, which is proportional to the phage concentration. Therefore, phages in more concentrated solutions are more prone to aggregation. The size of the aggregates formed at high phage concentrations was similar to the average size of the liposomes formed using the microfluidic device thereby limiting the likelihood of phage encapsulation in the formed liposomes. We found increasing the T3 phage titer did not yield correspondingly higher titers of encapsulated phages in liposomes (discussed later).

**Figure 4:**
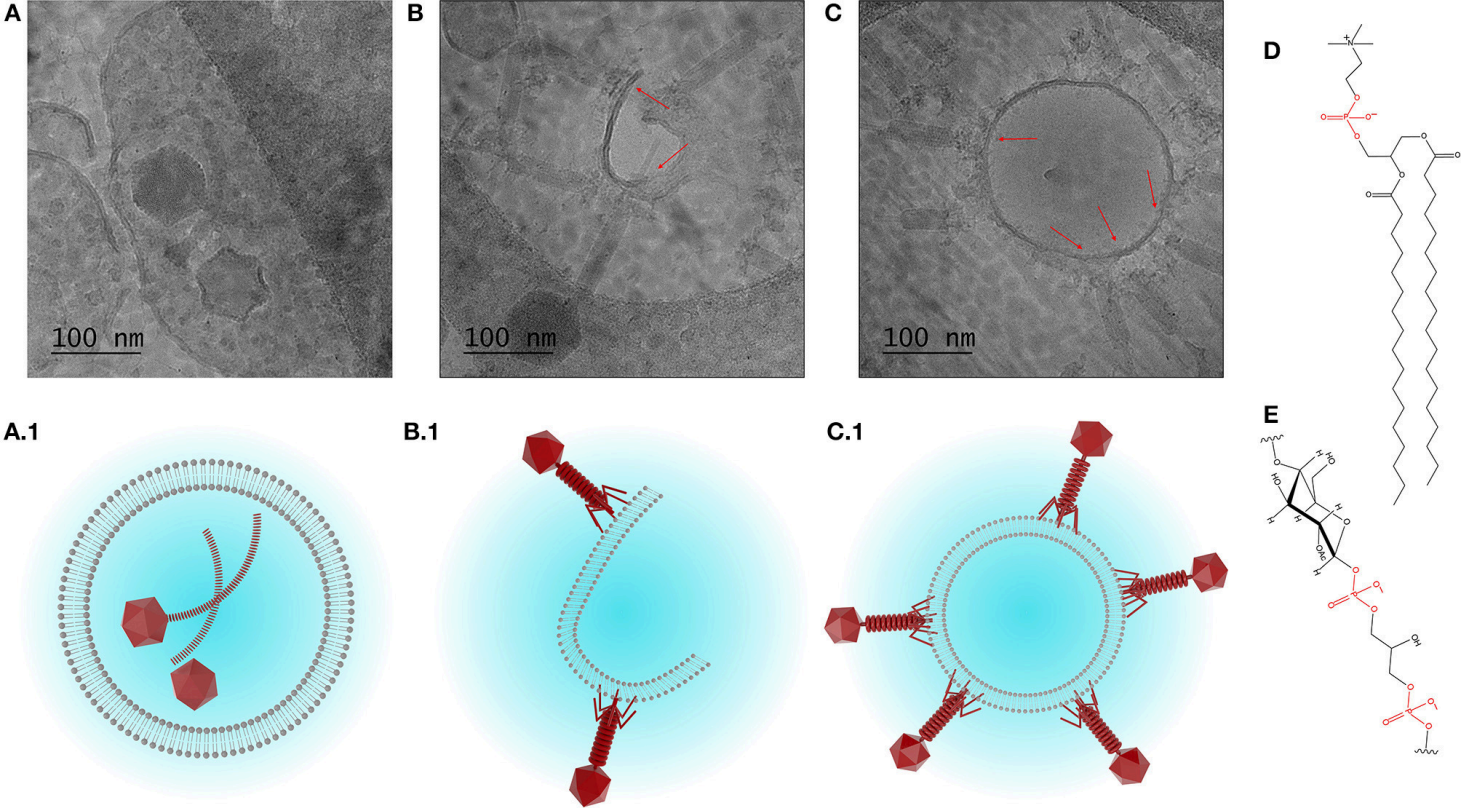
Cryo-TEM images showing **(A)** encapsulation of phage K, and **(B,C)** interaction between phage K tails and the outer leaflet of the bilayer membrane of formed liposomes (DSPC:cholesterol molar ratio was 5:1), some tails look contracted. **(A1–C1)** Schematized depiction showing encapsulation of phage K within the liposome (favored outcome) and interaction of phage K with the outer surface of unilamellar liposome membrane and lipid bilayer (unfavorable outcome). **(D)** Chemical structure of DSPC and **(E)** chemical structure of teichoic acid (phosphate groups highlighted in red).

The morphology and structure of the liposome-encapsulated phages were visualized using cryo-TEM (Figure 4). The liposomes were typically found to be unilamellar with a high proportion of empty liposomes and large numbers of unencapsulated phages (Figure S1). Unilamellar vesicles are usually prepared using significant energy inputs supplied by sonication or extrusion through a membrane at high pressure. Here, unilamellar vesicles were formed using a low energy method. The thickness of the bilayer membrane on cryo-TEM images is 4–5 nm, which corresponds to the thickness of the DSPC bilayer ~4.2 nm estimated from small angle neutron and X-ray scattering data (Kučerka et al., 2011).

Due to the relatively large head and long tail, phage K were found encapsulated only in large liposomes (~500 nm or bigger) which were formed using DSPC:cholesterol molar ratio of 5:1. Encapsulated liposomes contained typically one and sometimes two phages per liposome (Figure 4A). Verification of the encapsulation of phage K within the liposome was achieved through tilting of the sample during observation using tomography and reconstruction of the three dimensional structure of the liposome encapsulated phage (tomography reconstruction and extended looped video provided as Supplementary Materials). The tomography image shows layer-by-layer reconstruction of phage K located within the liposome. Multiple phage K tails were observed to frequently interact with the lipid bilayer and bind to the outside wall of the fully formed liposomes as well as lipid bilayer fragments (Figures 4B,C). Schematic representations of encapsulated and externally bound phages are also shown (Figures 4A.1–C.1).

**Figure 5:**
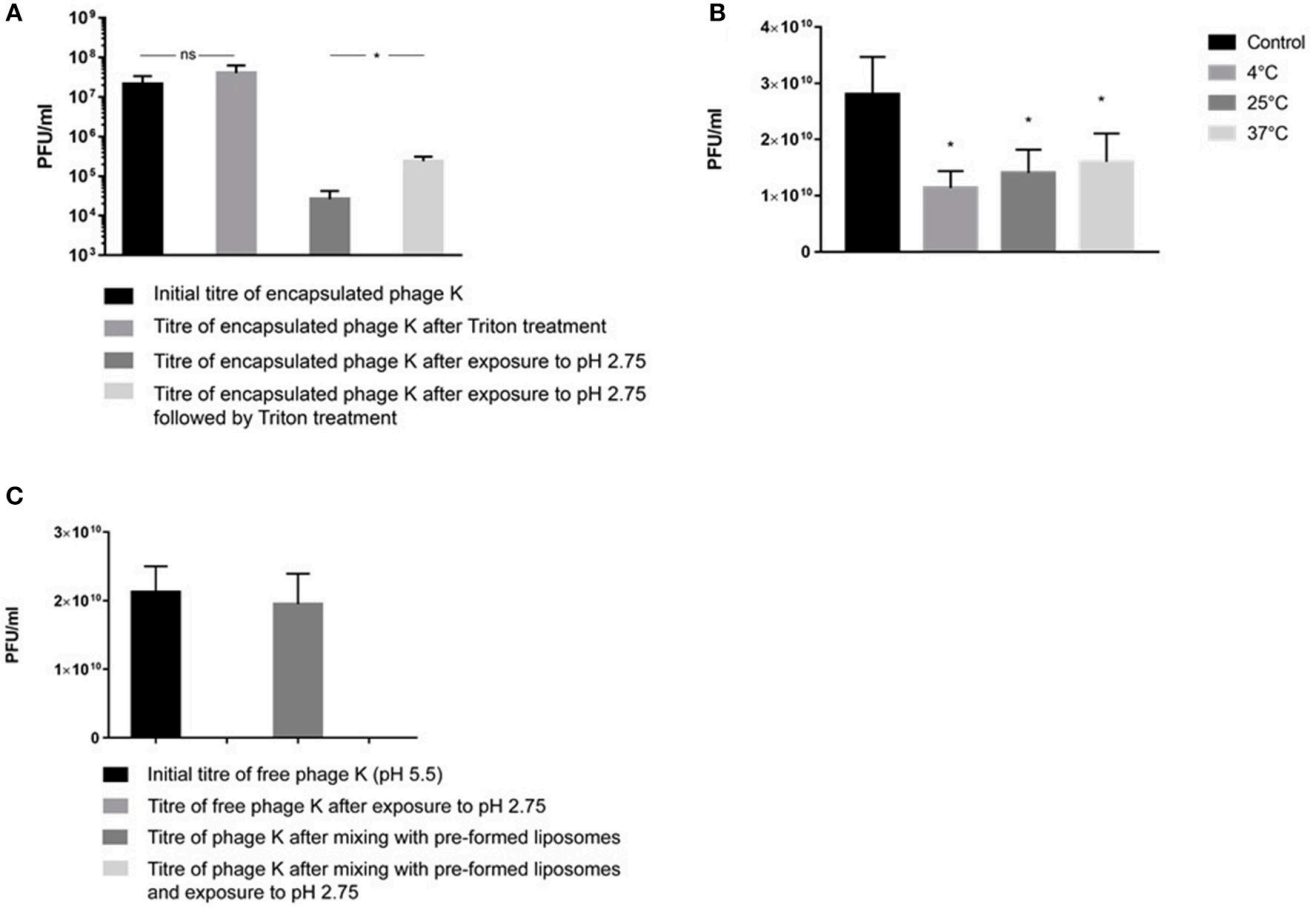
**(A)** *S. aureus* phage K encapsulation in DSPC:cholesterol liposomes. Phages were encapsulated at FRR of 2:1 at room temperature. Phage titer of liposome encapsulated phages prior to and after acid exposure at pH 2.75 (with and without Triton X-100 disruption). *Indicates significance (*p* < 0.05) using 2-sample *t*-test. **(B)** The titer of free phages in the supernatant before and after incubation with empty liposomes at 4, 25, and 37°C for 15 min. After incubation, liposomes were separated by centrifugation at 13,000 × *g* for 3 min and the phage titer in the supernatant was measured. The difference in the titer occurred due to phage adsorption onto the outer leaflet of bilayer membranes. The maximum phage adsorption was observed at 4°C, which caused the highest phage titer reduction in the supernatant at 4°C. The adsorption was still highly significant at 37°C. *Indicates significance (*p* < 0.05) using 2-sample *t*-test of each sample compared with controls. **(C)** pH stability for free (non-encapsulated) phage K exposed to pH 2.75 and free phage K incubated with pre-made empty liposomes for 15 min at 37°C and subsequently dialyzed at pH 2.75 for 60 min. Error bars represent one standard deviation.

Liposome suspensions containing encapsulated phage K prepared using the formulation with a DSPC:cholesterol molar ratio of 5:1 were tittered for free phages before [sample mean 2.1 × 10^7^ PFU/ml, 95% Confidence Interval (CI) 1.1 × 10^7^-3.2 × 10^7^ PFU/ml] and after disruption of the liposomes (sample mean 4 × 10^7^ PFU/ml, 95% CI 2.1 × 10^7^-5.9 × 10^7^ PFU/ml) following treatment with Triton X-100 to disrupt the liposomes (Figure 5A). Disruption of the liposomes did not result in a statistically significant change in measured phage titers (*p* = 0.068). The phages remained viable following treatment with Triton X-100 (this was also the case for T3 phages, data not shown). Liposome suspensions containing free and encapsulated K phages were then exposed to pH 2.75 to inactivate free and liposome bound phages. Acid exposure of liposomes (containing K phages at pH 5.5) resulted in a significant reduction in phage titer (sample mean 2.6 × 10^4^ PFU/ml, 95% CI 1.3 × 10^4^-4 × 10^4^ PFU/ml). Acid exposed liposomes were subsequently disrupted with Triton X-100 to release any viable K phages remaining inside the liposomes. Disruption of liposomes resulted in a significant increase in phage titer (sample mean 2.4 × 10^5^ PFU/ml, 95% CI 1.8 × 10^5^-3 × 10^5^ PFU/ml). The 95% CI for the increase in phage titer upon liposome disruption was 1.5 × 10^5^-2.7 × 10^5^ PFU/ml which is indicative of the yield of encapsulated phage K in the liposomes. It was not possible to quantify the loss (if any) in titer of encapsulated phage K due to acid exposure. When the same experiment was carried out with the pH inside the liposomes kept at pH 7.5 and liposomes subsequently exposed to pH 2.75, no viable phages were recovered. Encapsulating phage K using a pH 5.5 buffer allowed us to have a lower pH inside the liposome which reduced the pH gradient across the liposome membrane bilayer to less than ΔpH 3 and hence reduced the driving force for proton flux (Deamer, 1987). A reverse of this approach has been used previously to load liposomes with drugs in response to transmembrane pH gradients (Mayer et al., 1990). The reduction in phage titer following acid exposure to pH 2.75 may be attributed to inactivation of both free phages and phages externally bound to the liposomes which was confirmed separately (Figure 5C).

Empty liposomes prepared using the formulation with a DSPC:cholesterol molar ratio of 5:1 were gently mixed with free phage K for 15 min at 4, 25, and 37°C. A considerable amount of phages adsorbed to the outer surface of empty liposomes resulting in a significant decrease in phage titer of the supernatant for all investigated temperatures (Figure 5B). Exposure to pH 2.75 of the K phages bound to empty liposomes resulted in no recovery of viable phages (Figure 5C), suggesting that K phages bound to the outside of the liposomes were not protected from acid exposure and were inactivated. In addition, free phages present in solution without the presence of any liposomes were also completely inactivated after exposure to pH 2.75 (Figure 5C). On the other hand, phages truly encapsulated in liposomes were able to withstand acid exposure. This procedure provided a means of discriminating between free phages, phages externally bound to liposomes and phages actually encapsulated within the liposomes.

**Figure 6:**
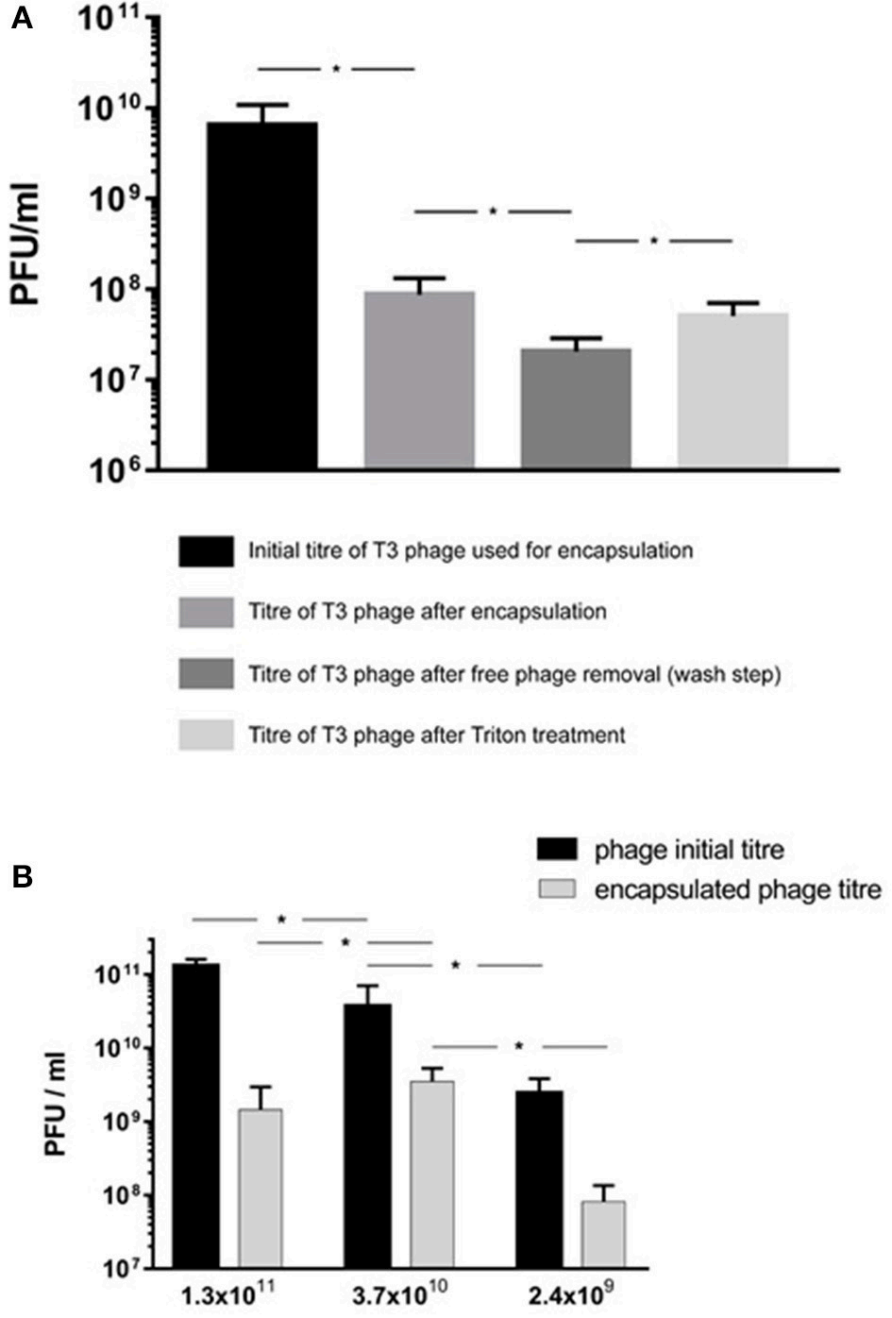
*E. coli* T3 phage encapsulation in DSPC:cholesterol liposomes. T3 phages were encapsulated at FRR of 2:1 at room temperature. Free phages were separated by centrifugation for 10 min at 13,000 × *g* and re-suspended in SM buffer, the process was repeated three times to remove unencapsulated phages. Liposomes were disrupted with Triton X-100 and phage titer assessed by plaque assay. **(A)** Phage T3 titers following encapsulation, after removal of unencapsulated free phages (following three wash steps) and after liposome disruption with Triton X-100. **(B)** Phage encapsulation yield using different initial phage titers and after encapsulation, free phage removal by centrifugation (as above) and then liposome disruption with Triton X-100. *Indicates significance (*p* < 0.05) using 2-sample *t*-test.

The encapsulation process resulted in a significant reduction in phage T3 titer upon exposure to IPA during the liposome formation process (Figure 6A). The phage titer fell from 2.4 × 10^9^ PFU/ml (95% CI 1.1 × 10^9^-3.7 × 10^9^ PFU/ml) to 8.7 × 10^7^ PFU/ml (95% CI 4.5 × 10^7^-1.3 × 10^8^ PFU/ml). Removal of free phages by centrifugation and resuspension in SM buffer resulted in phage T3 titer reduction from 8.7 × 10^7^ to 2.1 × 10^7^ PFU/ml (95% CI 1.3 × 10^7^-2.8 × 10^7^ PFU/ml). Disruption of the liposomes with Triton X-100 resulted in a significant increase in phage titer to 8.1 × 10^7^ PFU/ml (95% CI 3.1 × 10^7^-1.3 × 10^8^ PFU/ml) (Figure 6A). The difference in means was 6.1 × 10^7^ PFU/ml (95% CI 1 × 10^7^-1.1 × 10^8^ PFU/ml) which is an estimation of the phage T3 encapsulation yield.

Increasing free phage T3 titers used in the MF device for encapsulation from ~10^9^ to ~10^10^ PFU/ml resulted in a significant increase in encapsulated phage yield from 8.1 × 10^7^ PFU/ml (95% CI 3.1 × 10^7^-1.3 × 10^8^ PFU/ml) to 3.5 × 10^9^ PFU/ml (95% CI 1.9 × 10^9^-5.2 × 10^9^ PFU/ml) (Figure 6B). Increasing the free phage T3 titers further to ~10^11^ PFU/ml resulted in a lower encapsulated phage yield with sample mean falling to 1.5 × 10^9^ PFU/ml (95% CI 7.5 × 10^7^-2.8 × 10^9^ PFU/ml) (Figure 6B). The encapsulated phage yield was lower for the sample prepared using higher phage titer of ~10^11^ PFU/ml compared with ~10^10^ PFU/ml.

## Discussion

Micromixing of the lipid-containing organic phase with the aqueous buffer resulted in self-assembly of lipid bilayers in the collection channel of the MF device. This process is driven by hydrophobic effects in order to minimize entropically unfavorable interactions between the hydrophobic acyl chains and the surrounding aqueous buffer phase (Carugo et al., 2016). The lipid-in-alcohol stream was sheathed by a coaxial aqueous stream resulting in hydrodynamic flow focusing of the core fluid (Vladisavljevic et al., 2014). Microfluidic flow focusing permitted mixing of the two phases under low Reynolds number flow conditions (Re ~1) and diffusion dominated mass transfer (Peclet number <1) resulting in controlled production of nanoscale liposomes (Jahn et al., 2004). A 3D geometry of the MF device allowed radially symmetric interdiffusion of IPA-solvated lipids, alcohol, and water across the alcohol-water interface which offers advantages including low polydispersity and higher production rates (Hood et al., 2014). The cross-sectional area of the core solution may be tuned by adjusting the orifice size of the injection capillary and the FRR of the outer (aqueous) and inner (organic) phases (Jahn et al., 2010). At low Reynolds and Peclet numbers used in this work laminar flow conditions prevailed in the downstream capillary and liposome formation was dominated by diffusion rather than advection. The microfluidic micromixing process allowed predictable and repeatable mixing across fluid interfaces and the continuous synthesis of liposomes of narrow size distributions and controlled size which could be modulated by controlling FRR, the total flow rate and composition of the lipids. The addition of cholesterol makes the assembled bilayers more rigid and therefore, more energy and time is required for their closure (Sawant and Torchilin, 2012a). Liposome formation in microchannels at low temperatures has been shown to result in larger liposomes compared with those produced under identical conditions albeit at higher temperatures (Wi et al., 2012). Increasing the proportion of organic phase to aqueous phase results in a shallower lipid concentration gradient and has previously been shown to result in larger liposomes for the same formulation (Jahn et al., 2010; Leung et al., 2018). We employed a FRR of 2:1 and a low total flow rate of ~1 ml h^−1^ which resulted in liposome sizes of the same order as those reported elsewhere under similar hydrodynamic conditions (Wi et al., 2012; Carugo et al., 2016; Leung et al., 2018).

Evidence of disc-like non-equilibrium lipid aggregates in a region close to the immediate mixing zone of the two phases in a microfluidic channel was recently shown using cryo-ultramicrotomy and cryo-SEM (Jahn et al., 2013). Time is needed for lipid molecules to diffuse and aggregate; the concentration gradient of alcohol has a significant effect on the liposome formation process with gentle gradients (low FRR) favoring large liposome formation (Jahn et al., 2013). The closure of lipid bilayers may incidentally trap any bacteriophages in the vicinity of the enclosing lipid membrane and the number of phages encapsulated per vesicle is dictated by Poisson statistics. Parabolic velocity profile in pressure driven laminar flows results in the velocity decreasing toward the capillary wall which affects axial and radial concentration profiles and the distribution of liposome sizes formed. A number of factors may be responsible for the observed polydispersity of the liposomes formed (Figure 2). The rate of mass transfer of the lipids is affected by the velocity distribution along and across the channel resulting in variations in mixing times across and along the microcapillary affecting the size distribution of the liposomes formed. Our intention in this work was not to study these effects which have been considered in detail elsewhere (Fernandez-Puente et al., 1994; Zook and Vreeland, 2010; Carugo et al., 2016).

A number of previous studies have noted albeit briefly that encapsulation of phages in liposomes prepared using either the thin film hydration method or microfluidic micromixing resulted in empty liposomes and noted that phages were attached to the outside of the liposomes (Colom et al., 2015; Nieth et al., 2015a; Leung et al., 2018). However, to the best of our knowledge none of these studies went further to quantify and decouple the proportion of phages truly encapsulated in the liposomes and those that were externally bound to the lipid membranes. Previous studies have used high titers of phages (10^10^-10^11^ PFU ml^−1^) in order to achieve high reported yields of what are thought to be “encapsulated phages” (Colom et al., 2015; Chadha et al., 2017; Leung et al., 2018). However, consideration needs to be given to phage aggregation, which is more significant at higher phage concentrations. The resulting phage clusters may be too big to be physically encapsulated within formed liposomes. We found increasing the phage T3 titer from 10^9^ to 10^10^ PFU ml^−1^ initially increased the encapsulated phage yield. Increasing the phage titer further to 10^11^ PFU ml^−1^ resulted in lowering of the phage T3 encapsulation yield (Figure 6B). This may be attributed to the formation of large phage T3 aggregates at high phage titers that are either too big to encapsulate in the liposomes or diffuse too slowly and are not in the vicinity of closing bilayers and therefore are not trapped in the liposomes (Figure 3). Other studies have reported phage aggregation and formation of cluster rosettes (Serwer et al., 2007; Bourdin et al., 2014). Methods for dispersion of phage aggregates may help in this regard (Szermer-Olearnik et al., 2017).

Our results suggest that the actual encapsulation yield of *S. aureus* K phages inside the liposomes may be considerably overestimated unless externally bound phages are accounted for. *S. aureus* phage K may bind to the lipid bilayers due to electrostatic interactions between negatively charged phosphate groups on the phospholipid head (shown in red in Figure 4D) and positively charged residues on the tail fibers of the phages. This interaction resembles a reversible interaction between phages and their hosts via wall teichoic acids, e.g., glycopolymers present in Gram-positive bacterial cell membranes (Figure 4E) (Xia et al., 2011). The reported encapsulation efficiencies have ranged between 40 and 80% which for large tailed phages may be attributed to phages externally bound to the lipid bilayers (Colom et al., 2015; Chadha et al., 2017; Leung et al., 2018). The adsorption process is reversible leading to a dynamic equilibrium between bound and unbound phages (Baptista et al., 2008; Xia et al., 2011). In a previous study, a 60 min exposure of liposome encapsulated *Salmonella* phages (prepared using thin film hydration technique) to simulated gastric fluid (pH 2.8) resulted in a significant reduction in phage titer (by 4–5 orders of magnitude) which mirrors the results reported here (Figure 5A) (Colom et al., 2015). In an effort to minimize acid induced phage inactivation within the internal liposome compartment we reduced the transmembrane pH gradient between the liposome core and the external solution to minimize proton permeability across the DSPC-cholesterol lipid membrane (Biloti et al., 2003). Phage encapsulated liposomes exposed to pH 2.75 showed a significant fall in phage titer to 10^4^ PFU ml^−1^ with a statistically significant increase of ~1 log in phage K titer upon liposome disruption with Triton X-100 indicating that some or perhaps all encapsulated phages survived the acid treatment. The encapsulated phage K titer was however quite low ~10^5^ PFU ml^−1^. Following liposome formation external phages may subsequently bind to the outer liposome membrane. Cryo-TEM images suggested that only one or two phages were encapsulated in each liposome. A 1 log increase in phage titer upon liposome disruption suggests that removal of free phages and inactivation of bound phages followed by disruption of the liposomes provides a means for evaluating the titer of encapsulated phages.

We only observed phage K bound to the outside of the formed liposomes and not when they were encapsulated inside the aqueous core. This may be due to the rate of formation of liposomes being much faster than the rate of interaction of phage K with the formed bilayer structures. The considerably faster interdiffusion of small molecules e.g., IPA and water may result in liposome formation in a zone close to the organic-aqueous interface. We hypothesize that due to their considerably larger size and hence much slower diffusion, phages may not be able to get near the vicinity of a large majority of rapidly forming bilayer discs in time to be encapsulated when the bilayers close resulting in low phage encapsulation yield. Aggregation of phages even at low phage titers compounds this effect making it problematic. Future efforts need to consider strategies to overcome these inherent difficulties if microfluidic approaches are to be successfully employed to encapsulate large entities such as bacteriophages in nanostructured materials.

## Conclusions

We have demonstrated the controlled microfluidic production of liposomes with a mean size in the range of 100–300 nm. The size of the liposomes may be controlled by incorporating different amounts of cholesterol in a formulation containing the phospholipid DSPC and regulating the hydrodynamic conditions within the microfluidic micromixing device, in particular the FRR of aqueous to organic phases. We show that the yield of T3 encapsulated phages may be adversely affected by aggregation which limited the maximum attainable yield of encapsulated T3 phages ~10^9^ PFU/ml. *S. aureus* phage K was found to adsorb to the external lipid membranes resulting in large numbers of phages bound to the outside of the formed liposome instead of being trapped inside them. We developed a method that permitted inactivation of liposome-bound K phages whilst retaining the activity of phages encapsulated within the liposomes. This allowed estimation of the encapsulated phage K yield which was found to be low ~10^5^ PFU/ml. Inactivation of encapsulated phage K due to acid exposure cannot be ruled out and needs further work. Previous studies on tailed phage encapsulation in liposomes may therefore have overestimated the yield of encapsulated phages which may affect the efficacy of phage dose delivered at the site of infection. In the case of treatment of gastrointestinal infections externally bound phages may be inactivated due to the stomach acidity.

## Author contributions

SC, DM, and FM wrote the methodology. SC and DM conceived the research, performed the formal analysis, executed the investigation and wrote the original draft of the manuscript. SB carried out the CryoTEM microscopy. DM organized the project administration and funding. DM, GV, and SB reviewed and edited the manuscript. DM and GV oversaw the supervision and resources.

### Conflict of interest statement

The authors declare that the research was conducted in the absence of any commercial or financial relationships that could be construed as a potential conflict of interest.

## Supplementary material

Figure S1 Cryo-TEM images showing non-encapsulated *S. aureus* phage K and *E. Coli* T3 phages and empty unilamellar liposomes. **(a)** Phage K (yellow arrow) and a unilamellar liposome (red arrow) **(b)** T3 phages (yellow arrows), some of them with empty heads and unilamellar liposomes (red arrows). Images presented for formulation (DSPC:cholesterol molar ratio was 5:1), FRR 2:1.

Video S1 Visualization of *S. aureus* phage K encapsulation in liposomes by Cryo-TEM tomography - extended video.

Video S2 Visualization of *S. aureus* phage K encapsulation in liposomes by Cryo-TEM and tomography reconstruction.
