# Supplementary figures and images for: Nanoencapsulation of Bacteriophages in Liposomes Prepared Using Microfluidic Hydrodynamic Flow Focusing
Source: Front Microbiol. 2018 Sep 12;9:2172. doi: 10.3389/fmicb.2018.02172 (PMC6144953; doi:10.3389/fmicb.2018.02172)

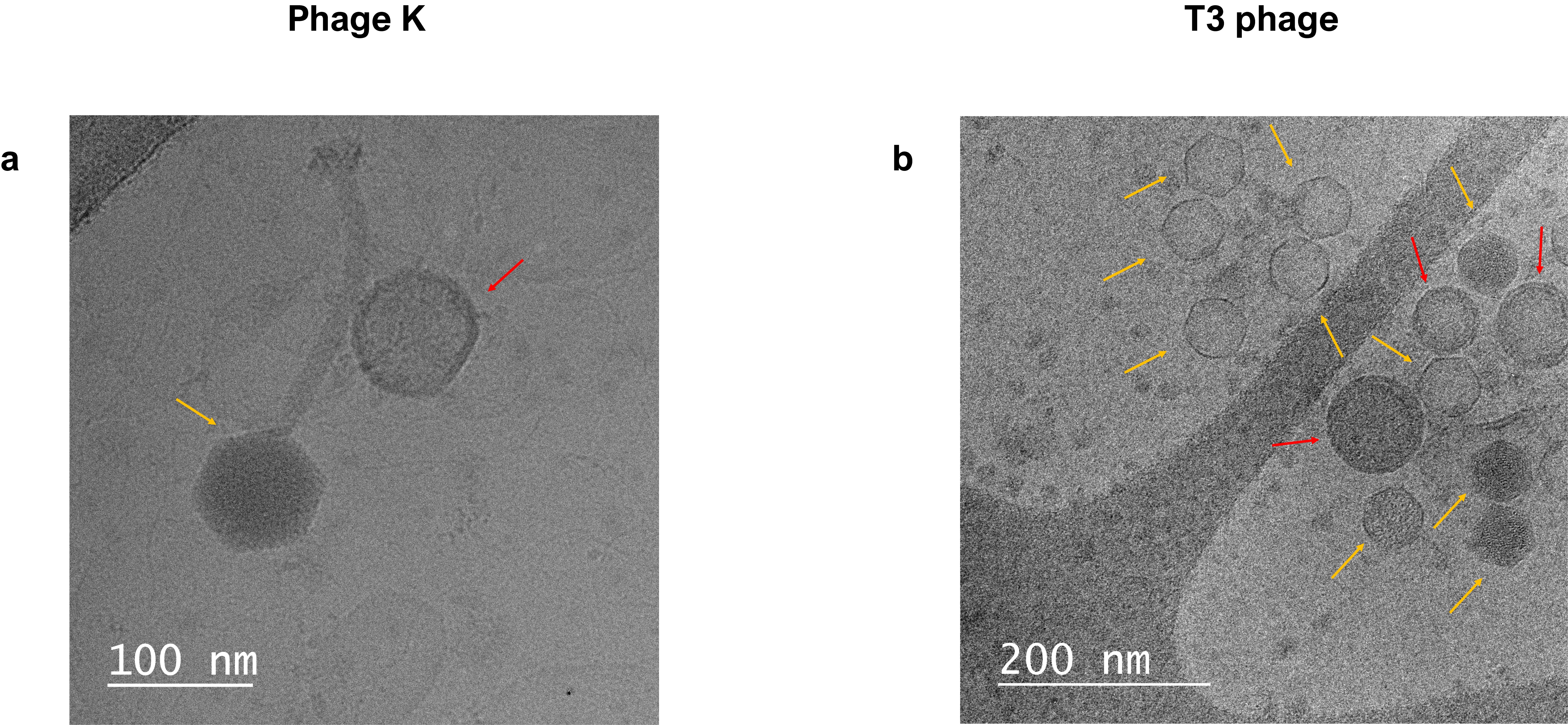

Supplement: Figure S1 — Cryo-TEM images showing non-encapsulated S. aureus phage K and E. Coli T3 phages and empty unilamellar liposomes. (a) Phage K (yellow arrow) and a unilamellar liposome (red arrow) (b) T3 phages (yellow arrows), some of them with empty heads and unilamellar liposomes (red arrows). Images presented for formulation (DSPC:cholesterol molar ratio was 5:1), FRR 2:1. [file Image_1.JPEG]
